# Artificial Intelligence Application in Assessment of Panoramic Radiographs

**DOI:** 10.3390/diagnostics12010224

**Published:** 2022-01-17

**Authors:** Łukasz Zadrożny, Piotr Regulski, Katarzyna Brus-Sawczuk, Marta Czajkowska, Laszlo Parkanyi, Scott Ganz, Eitan Mijiritsky

**Affiliations:** 1 Department of Dental Propaedeutics and Prophylaxis, Faculty of Dental Medicine, Medical University of Warsaw, 02-006 Warsaw, Poland; 2 Department of Dental and Maxillofacial Radiology, Faculty of Dental Medicine, Medical University of Warsaw, 02-091 Warsaw, Poland; piotr.regulski@wum.edu.pl; 3 Department of Comprehensive Dental Care, Faculty of Dental Medicine, Medical University of Warsaw, 02-091 Warsaw, Poland; kbrus@wum.edu.pl; 4 Department of Laryngology, Medical University of Silesia, 40-027 Katowice, Poland; 5 Department of Periodontology, Faculty of Dentistry, University of Szeged, 6720 Szeged, Hungary; parkanyilaci@gmail.com; 6 Department of Restorative Dentistry Rutgers, The State University of New Jersey, Newark, NJ 07103, USA; drganz@drganz.com; 7 Independent Researcher, Fort Lee, NJ 07024, USA; 8 Tel-Aviv Sourasky Medical Center, Department of Otolaryngology, Head and Neck and Maxillofacial Surgery, Sackler Faculty of Medicine, Tel Aviv 6139001, Israel; mijiritsky@bezeqint.net; 9 The Maurice and Gabriela Goldschleger School of Dental Medicine, Tel Aviv University, Tel Aviv 6997801, Israel

**Keywords:** AI, panoramic radiograph, screening, diagnosis, dentistry

## Abstract

The aim of this study was to assess the reliability of the artificial intelligence (AI) automatic evaluation of panoramic radiographs (PRs). Thirty PRs, covering at least six teeth with the possibility of assessing the marginal and apical periodontium, were uploaded to the Diagnocat (LLC Diagnocat, Moscow, Russia) account, and the radiologic report of each was generated as the basis of automatic evaluation. The same PRs were manually evaluated by three independent evaluators with 12, 15, and 28 years of experience in dentistry, respectively. The data were collected in such a way as to allow statistical analysis with SPSS Statistics software (IBM, Armonk, NY, USA). A total of 90 reports were created for 30 PRs. The AI protocol showed very high specificity (above 0.9) in all assessments compared to ground truth except from periodontal bone loss. Statistical analysis showed a high interclass correlation coefficient (ICC > 0.75) for all interevaluator assessments, proving the good credibility of the ground truth and the reproducibility of the reports. Unacceptable reliability was obtained for caries assessment (ICC = 0.681) and periapical lesions assessment (ICC = 0.619). The tested AI system can be helpful as an initial evaluation of screening PRs, giving appropriate credibility reports and suggesting additional diagnostic methods for more accurate evaluation if needed.

## 1. Introduction

**Figure 1:**
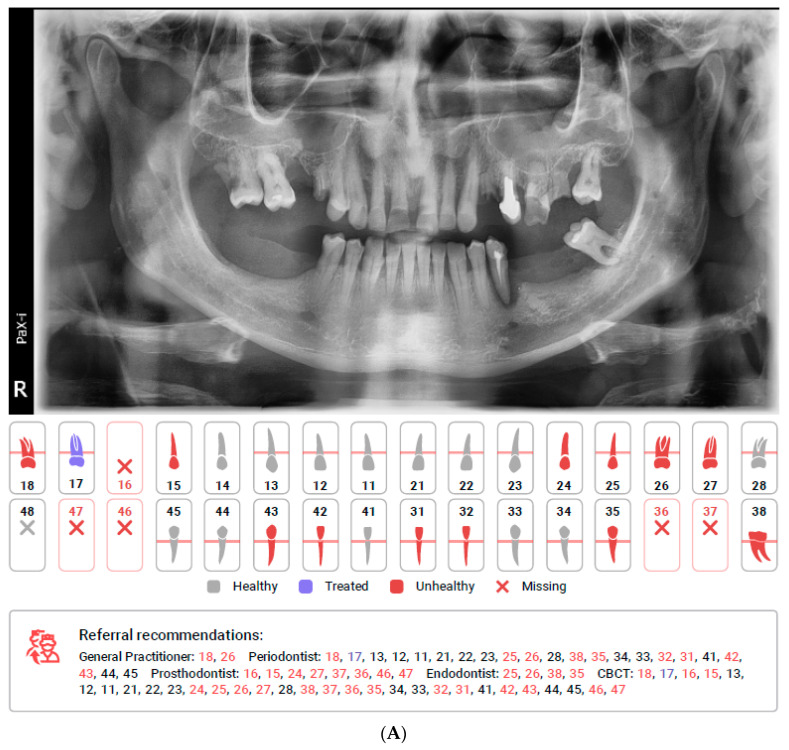

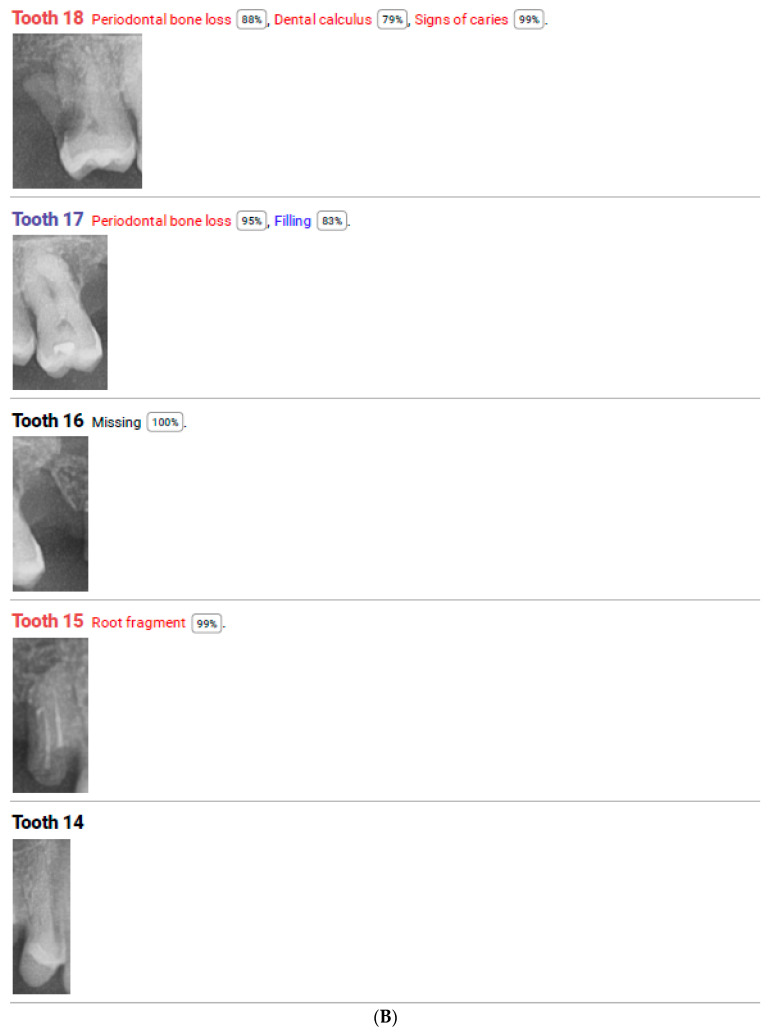
(**A**). First page of the DC report including simple diagram of teeth with a legend of findings and referral recommendations pointing specific specialists for specific teeth. (**B**). One of the following pages of the DC report including specific teeth captions and description with percent of accuracy.

Radiological examination is an essential part of patient management in modern dentistry. The panoramic radiograph (PR) is a common extraoral radiograph used to identify the hard tissues of the oral cavity and surrounding skeletal structures. Although resolution is not as detailed as intra-oral radiographs for examination of the teeth, many changes in calcification of the dental structures and in ossification of the surrounding bone can aid in the identification of dental diseases, such as caries (decay), periodontal bone loss, and bone lesions [1]. As far as cone-beam computed tomography (CBCT) systems are developed and becoming more and more popular for imaging comprehensive 3D volumetric information concerning oral soft tissues, bones, and teeth, PRs remain a very common initial X-ray and screening tool in the diagnostic process in dentistry [1,2,3,4,5,6,7,8,9,10,11,12]. However, although CBCT provides more data, the analysis is laborious and time-consuming [3,7,13]. PR analysis is faster than CBCT, but the accurate evaluation of all PR aspects still requires time and specialized knowledge. Thus, computer-aided systems have been developed to assist in medical and dental imaging diagnosis [14,15,16,17] and processing of the treatment [1,13,18,19]. One of the artificial intelligence (AI)-based systems based on the convolutional neural networks (CNN) is Diagnocat (LLC Diagnocat, Moscow, Russia). This is an online platform where different X-rays can be uploaded and analyzed by the algorithm. PR evaluation takes up to 2 min and the software generates a report (Figure 1). Such a report may focus the attention of the clinician on a specific problem or may be used as a communication aid with the patient to explain a required treatment. Moreover, the report contains suggestions for additional diagnoses, e.g., with use of CBCT or suggested consultations regarding specific sites with appropriate specialists. The aim of this study was to assess the reliability of Diagnocat software in the automatic evaluation of panoramic radiological images.

## 2. Materials and Methods

This retrospective research was performed following the principles of the Declaration of Helsinki and was approved by the Ethical Comity by the Medical University of Warsaw, Poland (Approval code: AKBE 221/2021). Thirty panoramic radiographs (PR) of 16 women and 14 men collected from the Dental and Maxillofacial Radiology Department, Medical University of Warsaw, Poland, taken from November 2019 to May 2021 were included in the study. Diagnostically acceptable or excellent quality radiographs, covering at least six teeth with the possibility of assessing the marginal and apical periodontium, were included. The exclusion criteria were: radiographs with unacceptable quality, containing severe artifacts, such as motion artifacts, shadow of the spine, or air projected on the region under assessment, radiographs containing developmental disorders. All PRs were listed and numbered. Then, all PRs were uploaded to the Diagnocat software (DC, Diagnocat LCC, Moscow, Russia) account, and the radiologic report of each was generated as the basis of automatic evaluation. The same PRs were manually evaluated by three independent dentists (evaluators) with 12, 15, and 28 years of experience in dentistry, respectively. One of the dentists (P.R) is experienced in dentomaxillofacial radiology. The missing teeth, presence of carries, dental fillings, prosthetic restorations (crowns or posts), endodontically treated teeth (with underfilled, overfilled or with inhomogeneous filling in the root canals), residual roots, periapical lesion (osteolytic, osteosclerotic or mixed), and periodontal bone loss were assessed. A special form was created to completed by each evaluator for each radiograph. Each evaluator assessed each radiograph independently and separately (without knowing the Diagnocat software evaluation). The reports were transferred to spreadsheets according to each pathology (category), tooth number, and evaluator. For each tooth, two possible values (presence of pathology or absence of pathology) were acceptable.

In order to assess the reliability of Diagnocat reports, they were compared with ground truth, obtained on the basis of analysis of three evaluators. If two or three evaluators agreed on the assessment, the diagnosis was considered as ground truth. Statistical analysis was done with SPSS Statistics software (IBM, Armonk, NY, USA). The sensitivity and specificity assessment was performed. Statistical analysis was performed for each pathology. Interclass correlation coefficient (ICC) analysis with a two-way mixed model was performed. It was assumed that ICC values greater than 0.75 would guarantee good reliability. In order to assess the interevaluator consistency, the ICC was also calculated.

The average time of evaluation was estimated for the creation of reports by different evaluators and AI software. 

## 3. Results

In total, 90 reports were created for 30 PRs. Overall numbers of evaluated pathologies are listed in the Table 1. The average time to prepare a single report was up to 2.0 min for DC and 8.5 min for evaluators.

The AI protocol showed very high specificity (above 0.9) in all assessments compared to ground truth except from periodontal bone loss. Sensitivity was very high (above 0.9) for the assessment of missing teeth and prosthetic restorations, and high (above 0.8) for dental fillings, endodontically treated teeth, residual roots, and periodontal bone loss. Low sensitivity was obtained for caries, periapical lesion, as well as over and underfilled canals assessment (see Table 1).

Statistical analysis showed high ICC (ICC > 0.75) for all interevaluator assessments, proving the good credibility of the ground truth and the reproducibility of the reports. The detailed results are shown in Table 2. 

The statistical assessment between ground truth and Diagnocat software results showed acceptable reliability (ICC > 0.75) for missing teeth, fillings assessment, prosthetic restoration, endodontically treated teeth (including under and overfilled canals), residual roots, and periodontal bone loss. Unacceptable reliability was obtained for caries assessment (ICC = 0.681) and periapical lesions assessment (ICC = 0.619) (see Table 3).

## 4. Discussion

**Figure 2:**
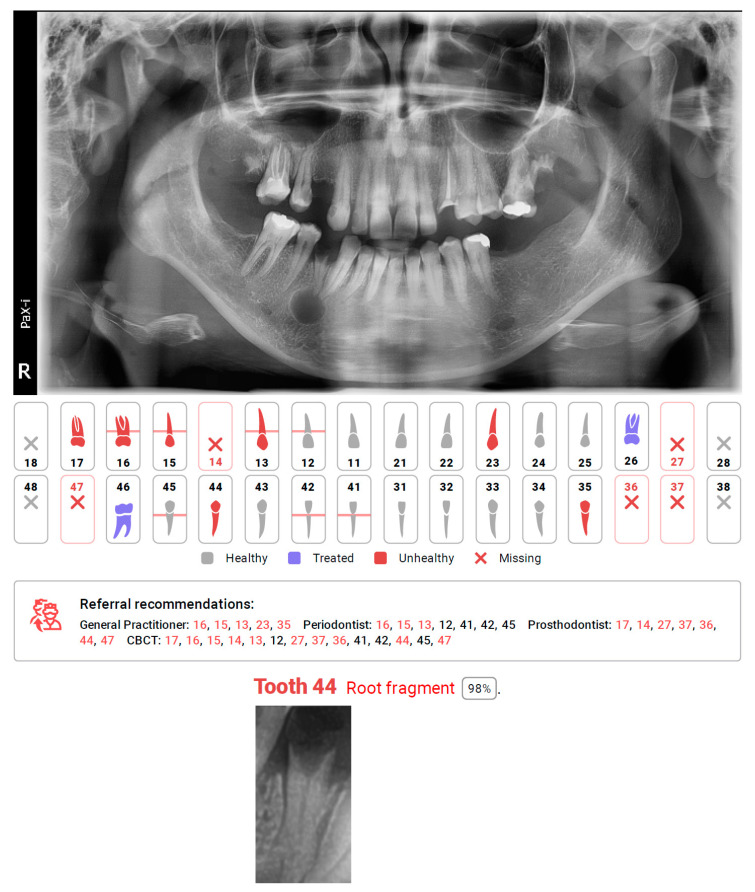
Diagnocat report, with missing detection of cyst connected with tooth 44, and automating caption of tooth recognized as a root fragment.

**Figure 3:**
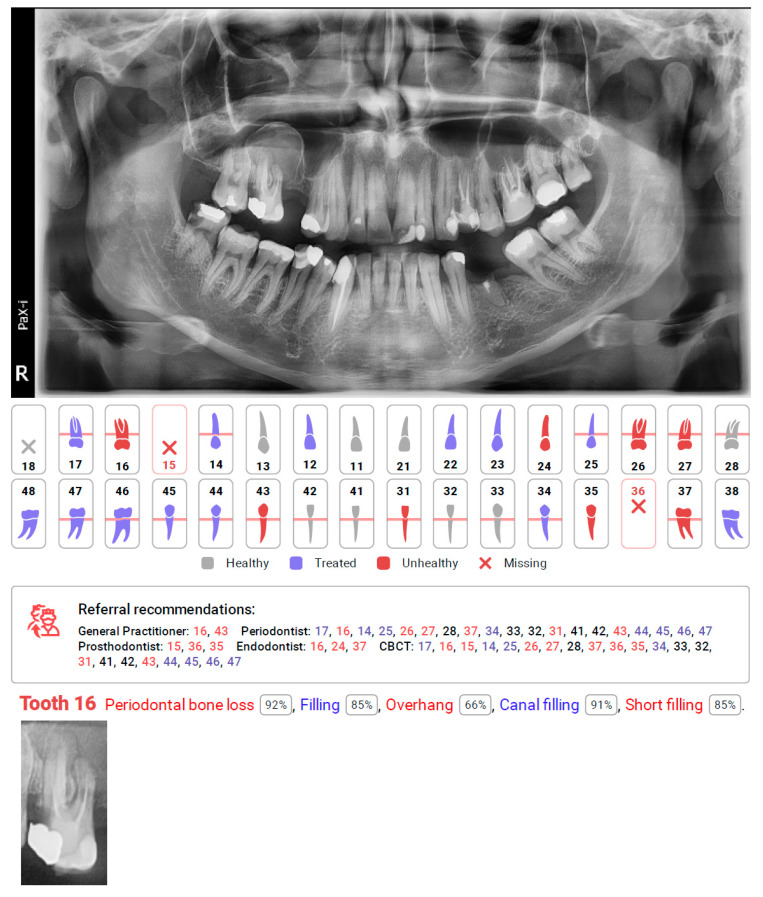
Diagnocat report, with missing detection of cyst in the right maxillary sinus in the region of tooth 16 and automating caption of tooth with detected other pathologies. Referral recommendations suggest additional CBCT diagnosis for this tooth as well as consultation with an endodontist.

The application of AI in medicine and dentistry has increased in recent years, which may be seen in the number of published studies [1,18,19,20,21,22,23,24,25,26,27]. The CNN based automatic protocol for X-ray evaluation used within this study presented high or very high sensitivity for dental fillings, endodontically treated teeth, residual roots, periodontal bone loss, missing teeth, and prosthetic restorations. Low sensitivity was obtained for periapical lesions, caries, as well as over and underfilled canals. Diagnocat did not detect any of the three periapical cysts, nor either of the two intramaxillary cysts or two broken endodontic instruments. However, the protocol did not name these specific pathologies. All teeth connected to these pathologies were marked as unhealthy and suggested for additional diagnostics using CBCT or referral for additional evaluation by a general practitioner (GP), endodontist (ED), or periodontist (PD) depending on the problem (see Figure 1 tooth 26, Figure 2 tooth 44, Figure 3 tooth 16). Our study shows the lowest reliability for apical periodontitis, which can be detected radiographically as periapical translucencies (a widened periodontal ligament or clearly detectable lesions). The detection and interpretation of a radiolucency in the periapical region is considered an important sign of periapical pathology. Although PRs represent the first, basic radiological overview X-rays, the detection of apical lesions on panoramic radiographs comes with limited sensitivity [28]. Nardi et al. in a retrospective study evaluated the diagnostic accuracy of panoramic radiographs in the detection of clinically/surgically confirmed asymptomatic apical lesions using CBCT imaging as the reference standard. Sensitivity, specificity, diagnostic accuracy, positive predictive value, and negative predictive value for panoramic radiographs with respect to CBCT imaging were analysed. Panoramic pictures showed good diagnostic accuracy, high specificity, and low sensitivity for the detection of endodontically treated apical periodontitis. The accuracy of detection also depends on the localisation and quality of the X-ray. The best identified apical lesions were located in the lower canine/premolar and molar areas, whereas the worst identified apical lesions were located in the upper/lower incisor area and upper molar area (anatomical conditions). These authors also found that the radiographic detection of apical lesions is subject to the large variation between examiners in terms of their experience. In our study, three experienced evaluators separately evaluated all the radiographic data from panoramic radiographs. In the inclusion criteria, we included the OPG quality criterium to limit the issue of localisation mentioned above. Among 805 assessments to reveal the presence or absence of the periapical lesions, obtained values of sensitivity and specificity were 0.390 and 0.981, respectively. 

The application of CNNs to assist in the detection of apical lesions could improve the ability to detect the apical lesions. The AI and deep learning protocol described by Ekert at al. [29] revealed that a moderately deep CNN trained on a limited amount of image data showed satisfying discriminatory ability to detect apical lesions on panoramic radiographs. The reference test was the majority vote of six independent examiners who detected apical lesions on an ordinal scale (0, no apical lesion; 1, uncertain apical lesion; 2, clearly detectable apical lesion, certain apical lesion) in comparison with the CNN protocol. The CNN based protocol revealed sensitivity and specificity values of 0.65 and 0.87, respectively. In molars, sensitivity was significantly higher than in other tooth types, whereas specificity was lower. The authors cautioned that the sensitivity of their system should be improved before clinical use. In our research, among 805 measurements, Diagnocat revealed unacceptable reliability with ICC = 0.619. The program failed to assess major osteolytic inflammatory lesions (e.g., cysts) in the periapical area. In a systematic review (search field 1862 titles, 50 studies included), the artificial intelligence models exhibited wide clinical applications in dentomaxillofacial radiology to identify maxillofacial pathologies including periodontitis/periapical disease. However, it is still necessary to further verify the reliability and applicability of the artificial intelligence models prior to transferring these models into clinical practice [14]. Regarding the diagnosis of periapical disease, Mol et al., as the pioneers of computer aided systems, concluded that interpretation could play an important role in the diagnosis of periapical bone lesions. Its objectivity and reproducibility can make it a valuable instrument for standardizing the diagnostic process [30]. It seems promising to use a more accurate radiologic tool as CBCT in artificial intelligence protocols. Orhan et al. used the same artificial intelligence system as we tested in our study, to detect periapical pathologies but on CBCT images. The images of 153 periapical lesions obtained from 109 patients were included in the study. The reliability of the artificial intelligence system in correctly detecting a periapical lesion was 92.8%. On the other hand, when analysing CBCT pictures by CNN: volumetric measurements of the lesions were similar to those with manual segmentation. There was no significant difference between the two measurement methods (*p* > 0.05). The authors concluded that artificial intelligence systems support the clinical diagnosis and can be useful for detecting apical lesions on CBCT. Under the conditions of these studies volume measurements performed by humans and by artificial intelligence systems were comparable to each other [31]. According to the literature, CBCT, as the modern radiologic tool, significantly increases the detection of periapical pathology compared to conventional periapical and panoramic radiographs [32,33]. Jae-Lee et al. evaluated the detection and diagnosis of three types of odontogenic cystic lesions, namely odontogenic keratocysts, dentigerous cysts, and periapical cysts, using dental panoramic radiography and CBCT based on a deep CNN. The pretrained model using CBCT images showed good diagnostic performance (sensitivity 96.1%, specificity 77.1%), which was significantly greater than that achieved by other models using panoramic images (sensitivity 88.2%, specificity 77.0%) (*p* = 0.014). The authors concluded that the CNN system trained with CBCT images obtained higher diagnostic performance than that trained with panoramic images [34]. Radiographic imaging for the diagnosis of caries lesions has been a part of clinical examinations for approximately a century. The value of radiography compared with a merely visual examination is especially emphasized in the diagnosis of caries lesions in clinically inaccessible surfaces, e.g., approximal. Detecting caries lesions on the radiographs can be questionable in some cases, depending on the experience of the person assessing the radiograph, localisation of the caries lesion, and type of radiograph (periapical, panoramic, bitewing, CBCT). Automated interpretation of the image with the aim to standardise diagnosis and optimise accuracy has been a research object in dentistry. Lee et al. evaluated the efficacy of CNN algorithms for detection of dental caries in periapical radiographs with rather high accuracy [35]. CNN systems were explored in the detection of caries lesions in bitewings. The research by Cantu et al. showed an accuracy of the system of 80%, while dentists’ mean accuracy was lower (71%). The AI system was significantly more sensitive than dentists, while its specificity was not significantly lower [36]. The neural networks used in detecting and diagnosing dental caries were also assessed by Prados-Privado et al. in a systematic review. The way in which each of the studies analysed caries (definition, type, tooth), as well as the parameters of each neural network (type of network, characteristics of the database, and results), were studied. Unfortunately, under the conditions of these studies and variable parameters assessed, the authors could not reach conclusive findings. Not all studies have detailed how detected caries are defined and not all of them specify the type of caries. Each study included in this review used a different neural network. All these variabilities complicated the conclusions about the subject, the reliability, or absence of a neural network in the detection and diagnosis of caries. Then, a comparison between the neural networking and clinical dental results are obligatory [37].

There are limitations in this study. The evaluated group of 30 PR is relatively small, although it provides data for appropriate statistical analysis. The second limitation is setting the ground truth as the basis of three evaluators’ reports. Furthers studies are needed in this field and authors of this research suggest involving a wider group of evaluators and performing analyses using larger samples.

## 5. Conclusions

Within the limitations of this retrospective study, we can draw the conclusion that the tested CNN based AI system can be helpful for an initial evaluation of screening PR for dental applications. Moreover, the report generated by the system refers to some potential pathologies to be evaluated by specific specialists or analysed with more accurate methods such as CBCT.

## Figures and Tables

**Table 1:** Sensitivity and specificity assessment of Diagnocat software.

Categories	Correctly Diagnosed (True Positive)	Mis-Diagnosed (False Negative)	Over-Diagnosed (False Positive)	Total Assessments	Sensitivity	Specificity
missing tooth	149	6	15	960	0.961	0.981
caries	89	111	11	805	0.445	0.982
filling	223	45	7	805	0.832	0.987
prosthetic restoration (crown or post)	44	2	4	805	0.957	0.995
endodontically treated tooth	95	14	4	805	0.872	0.994
underfilled canal	28	18	0	109	0.609	1.000
overfilled canal	5	6	0	109	0.455	1.000
inhomogeneous filling in canal	4	1	6	109	0.800	0.942
residual root	32	7	1	805	0.821	0.999
periapical lesion (osteolytic, osteosclerotic or mixed)	23	36	14	805	0.390	0.981
periodontal bone loss	189	47	87	805	0.801	0.847

**Table 2:** ICC for all interevaluator assessments (ICC >075).

Categories	ICC Interevaluator
missing tooth	0.977
caries	0.829
filling	0.928
prosthetic restoration (crown, post)	0.984
endodontically treated tooth	0.989
underfilled canal	0.924
overfilled canal	0.886
inhomogeneous filling in canal	0.834
residual root	0.969
periapical lesion (osteolytic, osteosclerotic or mixed)	0.903
periodontal bone loss	0.842

**Table 3:** ICC over ground truth for different evaluated objects.

Groups	ICC Diagnocat/Ground Truth
missing tooth	0.959
carries	0.681
filling	0.920
prosthetic restoration (crown, post)	0.968
endodontically treated tooth	0.948
underfilled canal	0.784
overfilled canal	0.752
inhomogeneous filling in canal	0.671
residual root	0.938
periapical lesion (osteolytic, osteosclerotic or mixed)	0.619
periodontal bone loss	0.764

## Data Availability

The data presented in this study are available on request from the corresponding author. The data are not publicly available due to privacy restrictions.
